# Disodium Cantharidinate and Vitamin B6 Injection Adjunct with Platinum-Based Chemotherapy for the Treatment of Advanced Non-Small-Cell Lung Cancer: A Meta-Analysis

**DOI:** 10.1155/2019/9386273

**Published:** 2019-03-12

**Authors:** Zhichao Wang, Fanchao Feng, Qi Wu, Yihan Jin, Cheng Gu, Yong Xu, Xianmei Zhou, Hailang He

**Affiliations:** ^1^Affiliated Hospital of Nanjing University of Chinese Medicine, Nanjing 210029, China; ^2^Department of Respiratory Medicine, Jiangsu Province Hospital of Chinese Medicine, Nanjing 210029, China

## Abstract

**Purpose:**

Disodium cantharidinate and vitamin B6 (DCVB6) injection is effective and widely used for the clinical treatment of non-small-cell lung cancer (NSCLC). This meta-analysis aimed to provide evidence-based medical data for clinical treatment with DCVB6 injection.

**Methods:**

We searched 7 medical databases up to January 2018 for all randomized controlled trials (RCTs) based on DCVB6 injection combined with chemotherapy in patients with NSCLC. A manual search in relevant journals and of relevant literature on other websites was also performed. Data extraction and quality assessment were conducted independently by two reviewers. Subsequently, a meta-analysis was conducted using RevMan 5.3 software. Pooled risk ratio (RR) with 95% confidence interval (CI) was used to evaluate dichotomous and continuous outcomes, respectively. The PROSPERO ID was CRD42018086377.

**Results:**

A total of 19 RCTs were included. The results of the meta-analysis indicated that the DCVB6 injection combined with chemotherapy was superior to chemotherapy alone regarding objective response rate (RR=1.58, 95% CI 1.40-1.79), Karnofsky performance score (RR=1.68, 95% CI 1.42-1.99), clinical symptom (RR=1.68, 95% CI 1.44-1.96), white blood cell toxicity (RR=0.36, 95% CI 0.27-0.49), platelet toxicity (RR=0.46, 95% CI 0.33-0.63), and vomiting (RR=0.50, 95% CI 0.37-0.67).

**Conclusions:**

The current evidence suggests that DCVB6 injection combined with chemotherapy could increase objective response rate and Karnofsky performance score, improve clinical symptoms, and reduce side effects caused by chemotherapy in patients with NSCLC. However, these results should be carefully interpreted due to the low-quality methodology and the small sample sizes of the trials, and our conclusions should be verified by high-quality, large-scale, double-blinded RCTs.

## 1. Introduction

Lung cancer is one of the most common malignant tumors and the leading cause of cancer death worldwide [1, 2]. Lung cancer brings a tremendous economic and social burden on both developing and developed countries [3]. Non-small-cell lung cancer (NSCLC) accounts for approximately 85% of all lung cancer cases [4].

Surgery is still the first choice and the most effective treatment for early-stage NSCLC. Unfortunately, more than half of NSCLC patients are initially diagnosed at an advanced stage [5]. Hence, they have to opt for other standard therapies, such as immunotherapy, targeted therapy, chemotherapy, or radiotherapy, for unresectable lesions. To date, platinum-based chemotherapy is still the dominant treatment for unresectable NSCLC due to its effectiveness in reducing tumor size [6]. However, some patients are unable to complete the recommended cycles of chemotherapy due to serious adverse events. Therefore, additional treatment strategies to enhance the clinical efficacy and alleviate the toxicity of chemotherapy are warranted.

Disodium cantharidinate and vitamin B6 (DCVB6) injection, a compound agent with pharmacological characteristics of both disodium cantharidinate and vitamin B6, is widely used as an adjuvant drug for patients with lung cancer undergoing chemotherapy in China [7, 8]. Per 10 ml of DCVB6 injection contains 0.1 mg disodium cantharidinate and 2.5 mg vitamin B6. The major ingredient of DCVB6 injection is cantharidinate, which is extracted from the Chinese blister beetle. It has been reported that cantharidinate can induce cell apoptosis, improve immunity, and inhibit the metastasis of tumor cells [9–11]. A number of clinical trials have revealed that DCVB6 injection combined with chemotherapy could increase the objective tumor response rate, improve performance status, and decrease the risk of adverse events compared with chemotherapy alone in patients with NSCLC. A previous meta-analysis indicated that DCVB6 injection combined with platinum-based chemotherapy might increase the effects and decrease the toxicity of chemotherapy for patients with NSCLC; however, only eight randomized controlled trials (RCTs) were included and the methodological quality of the included trials were inadequate [8]. Recently, new studies have evaluated the efficacy of DCVB6 injection combined with platinum-based chemotherapy for NSCLC. Therefore, we conducted this updated systematic review and meta-analysis to evaluate all related studies.

## 2. Materials and Methods

### 2.1. Publication Searching Strategy

The following major and authoritative English and Chinese electronic databases were searched up to January 2018: PubMed, Embase, the Cochrane Library, China National Knowledge Infrastructure Database, China Biological Medicine Database, WanFang Database, and China Science and Technology Journal Database. Two reviewers (Zhichao Wang and Fanchao Feng) independently searched for articles in these electronic databases using the following search strategy: (neoplasm [MeSH] OR lung neoplasm [MeSH] OR pulmonary neoplasms OR pulmonary neoplasm OR thoracic neoplasm OR pulmonary cancer OR lung cancer OR pulmonary carcinoma OR lung carcinoma OR NSCLC OR non-small-cell lung cancer) AND (disodium cantharidinate OR cantharidinate sodium OR disodium cantharidinate and vitamin B6 injection OR cantharidinate and vitamin B6 injection OR aiyishu). All of the retrievals were implemented using the MeSH and free words.

### 2.2. Inclusion Criteria

Eligible studies conformed to the following inclusion criteria: the disease was diagnosed and confirmed as NSCLC using histopathological or cytological diagnostic criteria; the TNM stage was advanced (III-IV); the type of study was randomized controlled trial (RCT) and the patients in each study were divided into two arms, the intervention for one arm was platinum-based chemotherapy alone, whereas the intervention for the other arm was platinum-based chemotherapy plus DCVB6 injection. In addition, at least one of the following outcomes must be contained in the reported data: (1) objective response rate (ORR); (2) Karnofsky performance score (KPS); (3) clinical symptom; (4) white blood cell (WBC) and platelet toxicity and vomiting incidence. Furthermore, the reported data needed to be sufficiently detailed to permit the calculation of the risk ratio (RR) and 95% confidence interval (CI) for each outcome.

### 2.3. Exclusion Criteria

Clinical trials were manually excluded if any of the following factors were identified: (1) patients with other malignancies were included in the trial; (2) interventions included other Chinese herbs or other traditional Chinese medicine (TCM) therapies; (3) duplicated articles; (4) the design scheme of the research was unclear or the data were incomplete.

### 2.4. Outcome Measures

Outcome measures included primary and secondary indices. ORR and KPS were primary outcomes. Clinical symptoms and adverse effects related to WBC and platelet toxicity and vomiting were regarded as the secondary indices of evaluation. ORR, formulated by the World Health Organization (WHO) scale [12], equals complete response (CR) + partial response (PR). The KPS [13] was employed in many of the included studies to investigate the performance status of patients by applying a 10-point change as the cutoff for improved or worse performance status. Therefore, we calculated improved performance status as the number of patients with improved performance status (>10-point increase) divided by the total number of patients. The 5-point WHO scale [12] was used to evaluate chemotherapy toxicity, and the rate of severe chemotherapy toxicity was defined as the number of patients with any severe toxicity (WHO grade 3 or 4) divided by the total number of patients in each treatment group (WHO grades 0, 1, 2, 3, and 4).

### 2.5. Data Extraction and Quality Assessment

Two investigators (Zhichao Wang and Fanchao Feng) independently reviewed the eligible studies and extracted the data. These results were cross-checked to ensure accuracy and reliability. The principal investigator (Xianmei Zhou) was consulted to resolve any discrepancies. The following information was collected from each article: (1) basic information such as language, year of publication, and name of the first author; (2) number of participants, gender, age, physical status, and TNM stage for each group; and (3) details of interventions and outcomes from each study. The methodological quality of the included RCTs was assessed independently by two reviewers (Zhichao Wang and Fanchao Feng) based on the criteria of the Cochrane Handbook for Systematic Reviews of Interventions Version 5.1.0. Briefly, the main questions about quality were (1) sequence generation; (2) allocation concealment; (3) blinding of participants, study personnel and outcome assessments; (4) incomplete outcome data, including baseline measurements before the intervention, effect parameters after intervention, and the dropout/exit rate (whether the dropout rate was less than 10%); and (5) selective outcome reporting. Each term was identified as having a low, unclear, or high risk of bias according to the criteria provided by the protocol. A widely used data abstraction form with a scoring system from 0 to 14 was applied to ensure the quality of the studies (Table S1).

### 2.6. Statistical Analysis

Review Manager version 5.3 (Cochrane Collaboration, Oxford, UK) was used to combine the data and perform the meta-analysis. Pooled RRs with 95% CIs were calculated to compare dichotomous and continuous variables respectively. The random effects model was applied if heterogeneity existed in pooled studies (*I*^2^ > 50%); otherwise, the fixed effect model was used. Statistical significance was considered as* P*<0.05. Funnel plots were employed to evaluate potential publication bias for primary outcomes if more than 10 studies were included for a meta-analysis [14]. Sensitivity analysis was employed by deleting individual trail at each turn to verify the robustness and reliability of the results.

## 3. Results

### 3.1. Retrieval Results

**Figure 1:**
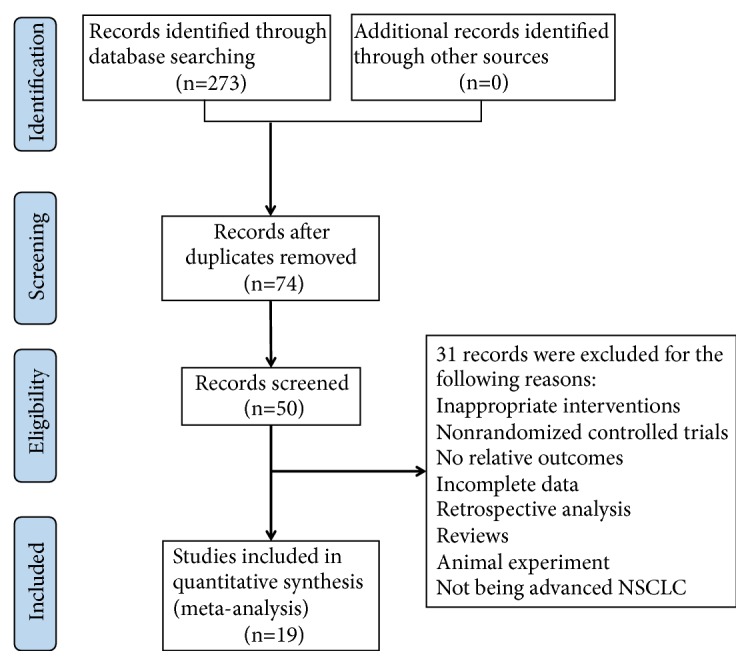
Flow diagram of the literature search process.

The initial search in electronic database identified 273 potentially relevant studies. A total of 74 records were identified after removing duplicates and screening the titles and abstracts. Thirty-one trials were excluded for the following reasons: animal experiments (n=2), reviews (n=4), inappropriate interventions (n=15), retrospective study (n=1), non-RCTs (n=1), no relative outcomes (n=4), incomplete data (n=3), and not regarding advanced NSCLC (n=1). Nineteen clinical trials [12–30] were finally included in this meta-analysis. A flowchart describing the literature search and study selection is shown in Figure 1.

### 3.2. Characteristics of Included Trials

Table 1 summarizes the main characteristics of the enrolled studies, including authors, year of publication, number of cases, the age of patients, patients' performance statuses, TNM stage, details of interventions, and outcomes. As shown, all studies were carried out in China and published in Chinese journals. The dosage of DCVB6 injection was 30-50 mL per day, and the duration of therapy was 10-15 days for 1-4 cycles by intravenous injection. The combination of gemcitabine plus cisplatin (GP) was the most common chemotherapy regimen [12, 13, 19, 23, 25, 27–29]; docetaxel plus cisplatin (DP), paclitaxel plus cisplatin (TP), navelbine plus cisplatin (NP), and pemetrexed plus cisplatin (AP) regimens were applied in 6 [13, 15, 18–21], 4 [16, 17, 19, 26], 3 [14, 19, 30], and 1 [24] studies, respectively. All the NSCLC patients enrolled in the included studies were at an advanced TNM stage.

### 3.3. Methodological Bias of the Included Studies

**Figure 2:**
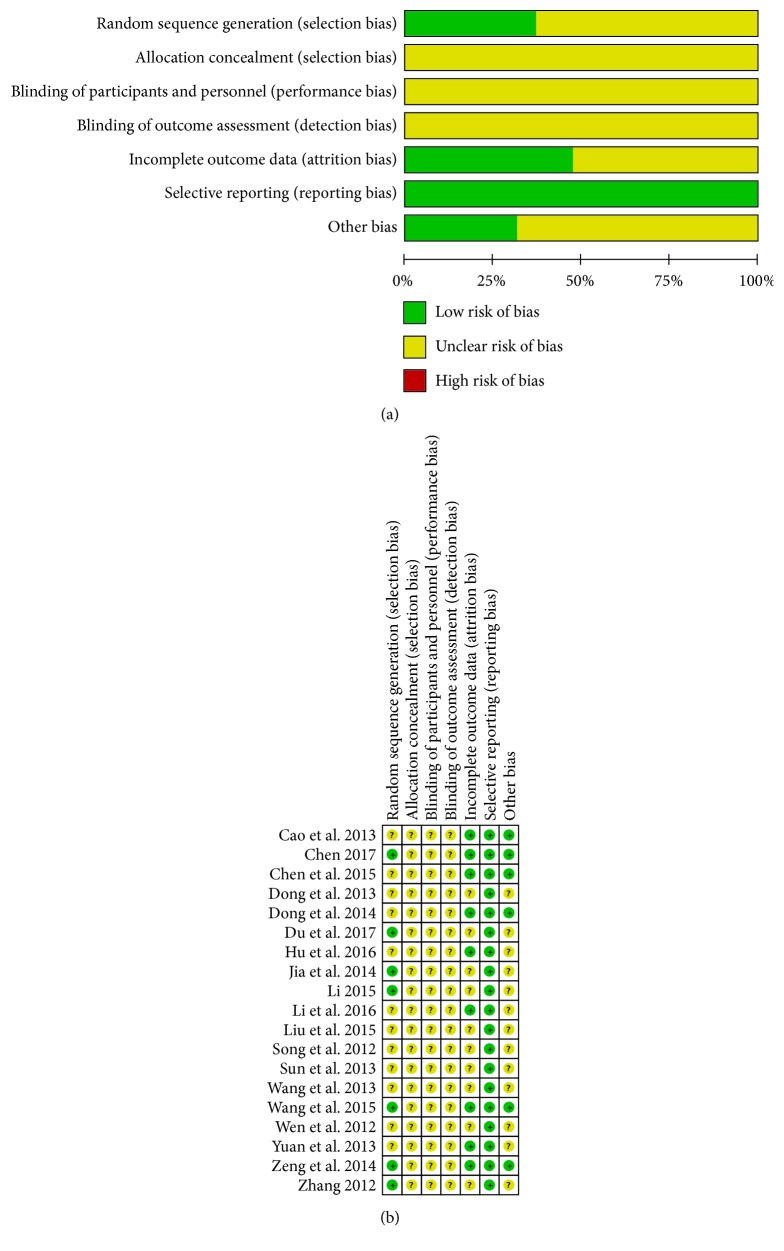
Risk of bias graph (a) and risk of bias summary (b).

All of the included trials mentioned randomization; however, only seven [17, 20, 21, 24, 26, 29, 30] described the specific methods of randomization. Six [17, 20, 21, 26, 29, 30] trials were randomized by using random number tables to generate a sequence. Although we tried to contact the original authors by phone or e-mail, we were unable to contact twelve [12–16, 18, 19, 22, 23, 25, 27, 28] of them. None of the trials mentioned allocation concealment methods. The blinding procedure was not mentioned in any of the studies suggesting that there were selection and high implementation biases. None of the trials showed results substantiating the integrity of their data. Selective reporting did not appear in all of the studies, and other biases were not clear. Detailed information on the methodological quality of the included studies is listed in Figure 2. Moreover, the individual values of each methodological score are available in Table 1.

### 3.4. Meta-Analysis for ORR

**Figure 3:**
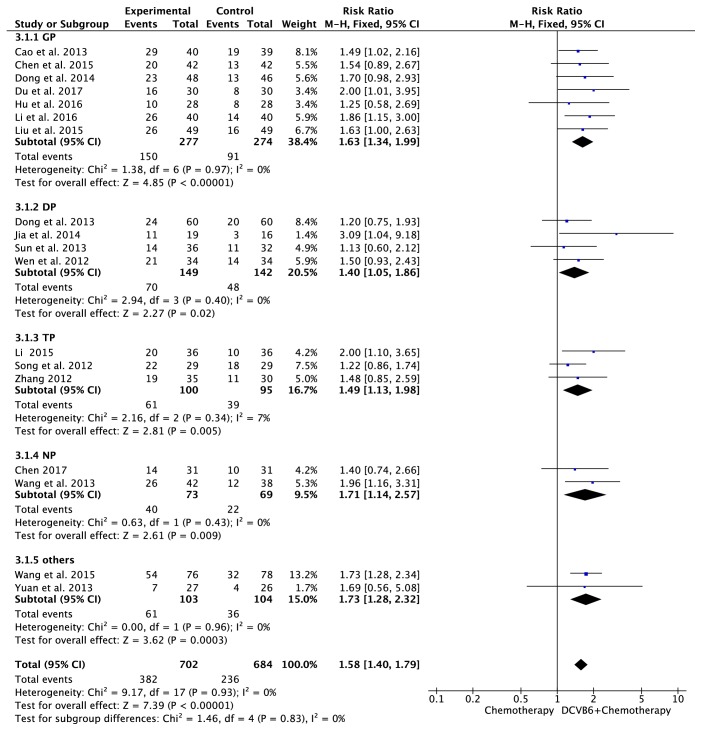
Forest plot of ORR.

Eighteen trials [12–19, 21–30] including 1386 cases reported ORR (Figure 3). There was no significant heterogeneity among the trials (*I*^2^=0%, *P*=0.93); therefore, the fixed-effects model was applied for the analysis. The results of the meta-analysis showed that the combination treatment of DCVB6 injection and platinum-based chemotherapy significantly improved the ORR of patients with NSCLC compared to chemotherapy alone (RR=1.58, 95% CI 1.40-1.79, *P*<0.00001). In the subgroup analysis, the pooled RR of ORR was 1.63 (95% CI 1.34-1.99, *P*<0.00001) for the GP regimen, 1.40 (95% CI 1.05-1.86, *P*=0.02) for the DP regimen, 1.49 (95% CI 1.13-1.98, *P*=0.005) for the TP regimen, 1.71 (95% CI 1.14-2.57, *P*=0.009) for the NP regimen, and 1.73 (95% CI 1.28-2.32, *P*=0.0003) for the other regimens.

### 3.5. Meta-Analysis for KPS

**Figure 4:**
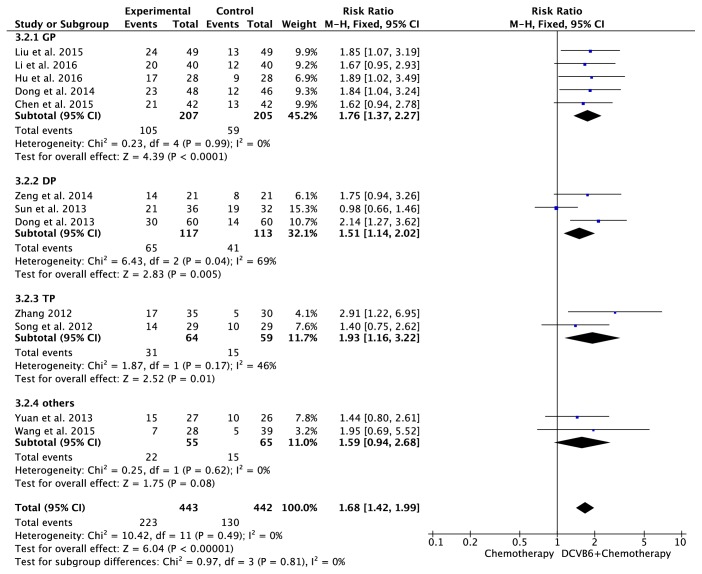
Forest plot of KPS.

Twelve trials [13, 16–20, 22–25, 27, 28] including 885 cases reported KPS (Figure 4). There was no significant heterogeneity among the trials (*I*^2^=0%, *P*=0.49); therefore, the fixed-effects model was applied for the analysis. The results of the meta-analysis showed that the combination treatment of DCVB6 injection and platinum-based chemotherapy significantly improved the KPS of patients with NSCLC compared to chemotherapy alone (RR=1.68, 95% CI 1.42-1.99, *P*<0.00001). In the subgroup analysis, the pooled RR of KPS was 1.76 (95% CI 1.37-2.27, *P*<0.0001) for the GP regimen, 1.51 (95% CI 1.14-2.02, *P*=0.005) for the DP regimen, 1.93 (95% CI 1.16-3.22, *P*=0.01) for the TP regimen, and 1.59 (95% CI 0.94-2.68, *P*=0.08) for the other regimens.

### 3.6. Meta-Analysis for Clinical Symptom

**Figure 5:**
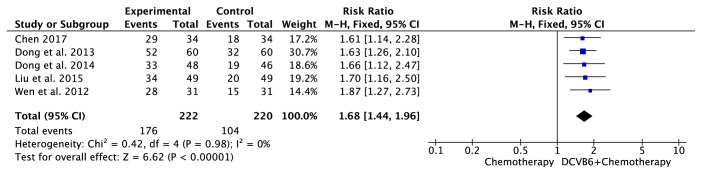
Forest plot of clinical symptom.

Five trials [13, 15, 22, 25, 30] including 442 cases reported clinical symptom (Figure 5). There was no significant heterogeneity among the trials (*I*^2^=0%, *P*=0.59); therefore, the fixed-effects model was applied for the analysis. The results of the meta-analysis showed that the combination treatment of DCVB6 injection and platinum-based chemotherapy significantly improved the clinical symptoms of patients with NSCLC compared to chemotherapy alone (RR=1.68, 95% CI 1.44-1.96, *P*<0.00001).

### 3.7. Meta-Analysis for Chemotherapy Toxicity

#### 3.7.1. WBC Toxicity

**Figure 6:**
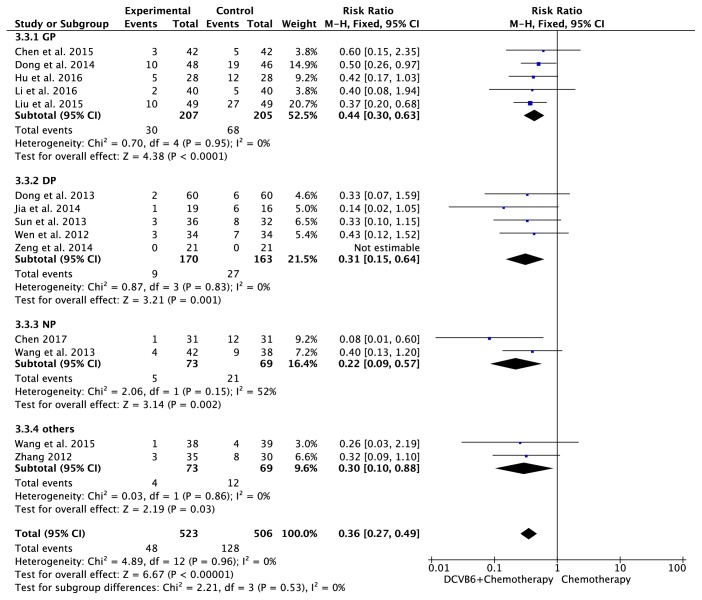
Forest plot of WBC toxicity.

There were 14 trials [13–15, 17, 18, 20–25, 27, 28, 30] including 1029 patients with WBC toxicity (Figure 6). The heterogeneity test indicated homogeneity (*I*^2^=0%, *P*=0.96), and the fixed-effects model was applied in this pooled analysis. Meta-analysis revealed that DCVB6 injection treatment significantly reduced the incidence of WBC toxicity compared to the control group (RR=0.36, 95% CI 0.27-0.49, *P*<0.00001). In the subgroup analysis, the pooled RR of WBC toxicity was 0.44 (95% CI 0.30-0.63, *P*<0.0001) for the GP regimen, 0.31 (95% CI 0.15-0.64, *P*=0.001) for the DP regimen, 0.22 (95% CI 0.09-0.57, *P*=0.002) for the NP regimen, and 0.30 (95% CI 0.10-0.88, *P*=0.03) for the other regimens.

#### 3.7.2. Platelet Toxicity

**Figure 7:**
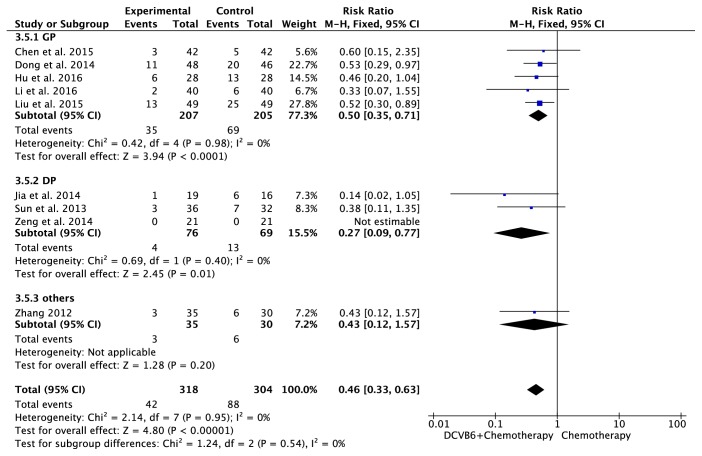
Forest plot of platelet toxicity.

There were 9 trials [17, 18, 20–23, 25, 27, 28] including 622 patients with platelet toxicity (Figure 7). The heterogeneity test indicated homogeneity (*I*^2^=0%, *P*=0.95), and the fixed-effects model was applied in this pooled analysis. Meta-analysis revealed that DCVB6 injection treatment significantly reduced the incidence of platelet toxicity compared to the control group (RR=0.46, 95% CI 0.33-0.63, *P*<0.00001). In the subgroup analysis, the pooled RR of platelet toxicity was 0.50 (95% CI 0.35-0.71, *P*<0.0001) for the GP regimen, 0.27 (95% CI 0.09-0.77, *P*=0.01) for the DP regimen, and 0.43 (95% CI 0.12-1.57, *P*=0.20) for the other regimens.

#### 3.7.3. Vomiting

**Figure 8:**
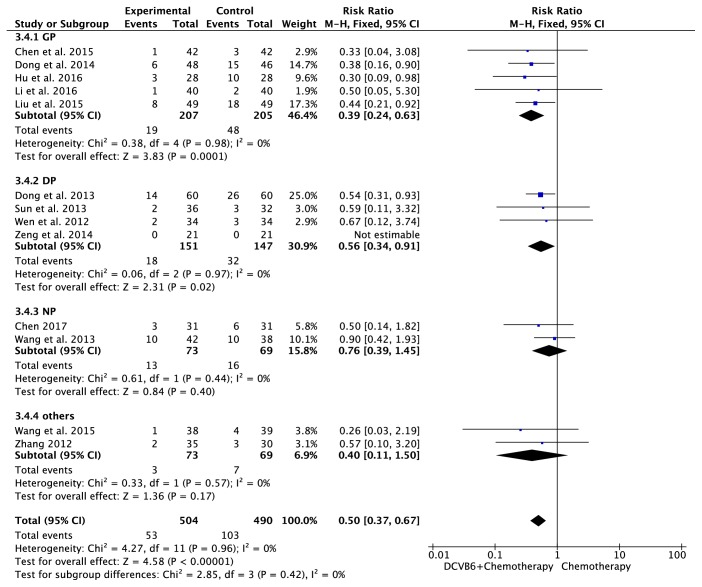
Forest plot of vomiting.

There were 13 trials [13–15, 17, 18, 20, 22–25, 27, 28, 30] including 994 patients with vomiting (Figure 8). The heterogeneity test indicated homogeneity (*I*^2^=0%, *P*=0.96), and the fixed-effects model was applied in this pooled analysis. Meta-analysis revealed that DCVB6 injection treatment significantly reduced the incidence of vomiting compared to the control group (RR=0.50, 95% CI 0.37-0.67, *P*<0.00001). In the subgroup analysis, the pooled RR of vomiting was 0.39 (95% CI 0.24-0.63, *P*=0.0001) for the GP regimen, 0.56 (95% CI 0.34-0.91, *P*=0.02) for the DP regimen, 0.76 (95% CI 0.39-1.45, *P*=0.40) for the NP regimen, and 0.40 (95% CI 0.11-1.50, *P*=0.17) for the other regimens.

### 3.8. Subgroup Analysis of Primary Outcomes

#### 3.8.1. Low versus High Dose of DCVB6 Injection

**Figure 9:**
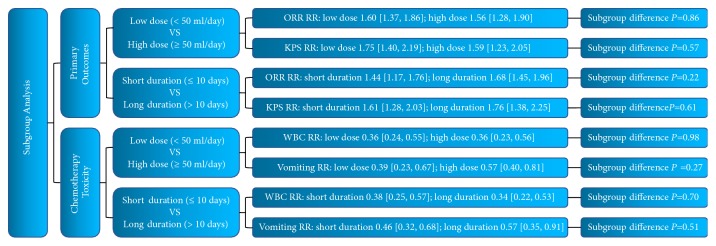
Subgroup analysis of primary outcomes and chemotherapy toxicity.

The median dose of DCVB6 injection was 50 ml/day. Of the 18 RCTs reporting ORR, 10 trials [12, 16, 17, 19, 21, 23–25, 27, 28] were included in the low dose of DCVB6 injection and 8 trials [13–15, 18, 22, 26, 29, 30] were in the subgroup of high dose (Figure S1). Of the 12 RCTs reporting KPS, 8 trials [16, 17, 19, 23–25, 27, 28] were included in the low dose of DCVB6 injection and 4 trials [13, 18, 20, 22] were in the subgroup of high dose (Figure S2). There was no differential effect of DCVB6 injection on ORR and KPS between the two subgroups (Figure 9).

#### 3.8.2. Short versus Long Duration of DCVB6 Injection

The median duration of DCVB6 injection was 10 days. Of the 18 RCTs reporting ORR, 8 trials [13, 15, 18, 22, 23, 27, 29, 30] were included in the short duration of DCVB6 injection and 10 trials [12, 14, 16, 17, 19, 21, 24–26, 28] were in the subgroup of long duration (Figure S3). Of the 12 RCTs reporting KPS, 5 trials [13, 18, 22, 23, 27] were included in the short duration of DCVB6 injection and 7 trials [16, 17, 19, 20, 24, 25, 28] were in the subgroup of long duration (Figure S4). There was no differential effect of DCVB6 injection on ORR and KPS between the two subgroups (Figure 9).

### 3.9. Subgroup Analysis of Chemotherapy Toxicity

#### 3.9.1. Low versus High Dose of DCVB6 Injection

The median dose of DCVB6 injection was 50 ml/day. Of the 14 RCTs reporting WBC toxicity, 7 trials [17, 21, 23–25, 27, 28] were included in the low dose of DCVB6 injection and 7 trials [13–15, 18, 20, 22, 30] were in the subgroup of high dose (Figure S5). Of the 13 RCTs reporting vomiting, 6 trials [17, 23–25, 27, 28] were included in the low dose of DCVB6 injection and 7 trials [13–15, 18, 20, 22, 30] were in the subgroup of high dose (Figure S6). There was no differential effect of DCVB6 injection on WBC toxicity and vomiting between the two subgroups (Figure 9).

#### 3.9.2. Short versus Long Duration of DCVB6 Injection

The median duration of DCVB6 injection was 10 days. Of the 14 RCTs reporting WBC toxicity, 7 trials [13, 15, 18, 22, 23, 27, 30] were included in the short duration of DCVB6 injection and 7 trials [14, 17, 20, 21, 24, 25, 28] were in the subgroup of long duration (Figure S7). Of the 13 RCTs reporting vomiting, 7 trials [13, 15, 18, 22, 23, 27, 30] were included in the short duration of DCVB6 injection and 6 trials [14, 17, 20, 24, 25, 28] were in the subgroup of long duration (Figure S8). There was no differential effect of DCVB6 injection on WBC toxicity and vomiting between the two subgroups (Figure 9).

### 3.10. Analysis of Publication Bias

**Figure 10:**
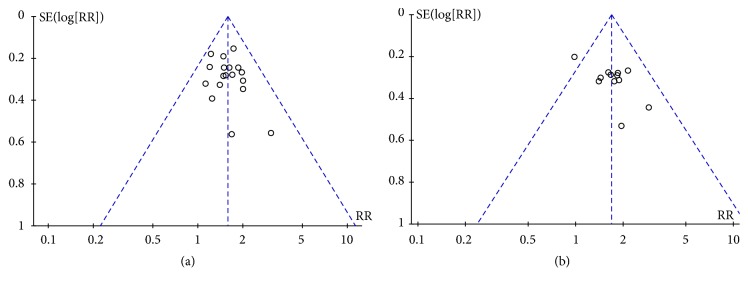
Funnel plots for assessing publication bias. (a) ORR; (b) KPS.

Funnel plots were plotted to identify potential publication bias among the included studies. The funnel plots were asymmetric in the studies regarding ORR and KPS (Figure 10), indicating that there was a potential risk for publication bias. Not only would publication bias cause asymmetry in funnel plots, but clinical or methodological heterogeneity between studies might also affect the shape of funnel plots.

## 4. Discussion

Chemotherapy combined with TCM has become a new model in the systemic treatment of lung cancer. A large number of clinical studies have proven that disodium cantharidinate not only has an antitumor effect but also reduces the side effects caused by chemotherapy and radiotherapy and improves quality of life. According to previous studies, disodium cantharidinate has a variety of pharmacological effects: (1) killing and inhibiting tumor cells directly by reducing DNA and RNA precursor substance intake to inhibit nucleic acid metabolism in tumor cells, decreasing the intake of amino acids to inhibit the synthesis of vital proteins in tumor cells, and inducing tumor cell apoptosis; (2) significantly enhancing the immune function of patients by stimulating macrophages, lymphocytes, and polymorphonuclear leukocytes to produce interleukins, changing T cell subsets to promote an effective antitumor immune response, and enhancing phagocytosis of tumor cells; (3) increasing WBC count leading to leukocytosis by promoting the differentiation of bone marrow hematopoietic stem cells into granulocyte-monocyte progenitor cells and shortening the time of leukocyte maturation; and (4) an analgesic effect thereby significantly relieving the pain of cancer after medication [31]. In the last decade, studies of the anticancer mechanism of disodium cantharidinate found that it can promote lymphocyte secretion of interleukin-2 cytokines to enhance immune function, reduce side effects of chemotherapy, and improve the efficacy of chemotherapy [32]. Disodium cantharidinate also inhibits tumor metastasis by inhibiting the secretion of interleukin-8 and inhibiting neovascularization [33]. Vitamin B6 is involved in amino acid metabolism by promoting the decarboxylation of amino acids into *γ*-aminobutyric acid, thereby inhibiting the excitability of the central nervous system to reduce nausea and vomiting caused by chemotherapy. In addition, vitamin B6 can prevent leukopenia, promote cell growth, and reduce anemia and chemotherapy-induced side effects. In summary, the multipharmacological effects of DCVB6 injection play a vital role as an adjuvant to chemotherapy.

Although a few similar studies have been published in China previously, their low quality and small sample sizes do not provide strong evidence-based medical data. In this study, we selected 19 high-quality RCTs including 1428 NSCLC patients which ensured an adequate sample size for meta-analysis. To further ensure the quality of this study, we included the most recently published studies. We found that DCVB6 injection combined with chemotherapy can affect ORR, improve KPS, and reduce chemotherapy toxicity compared with conventional chemotherapy. Subgroup analysis demonstrated DCVB6 injection combined with GP regimen was more effective. In addition, there was no differential effect of DCVB6 injection for different dose and different duration through subgroup analysis. However, this study still has several shortcomings. The quality of the meta-analysis depends on the quality of the included trials. First, the trials included in this meta-analysis were all carried out in China with small sample sizes; therefore, the clinical trial design and implementation will inevitably be flawed. Second, the quality of the trials was not sufficiently high. There were no large-scale multicenter clinical RCTs, and most of the trials only referred to randomized grouping but did not specify the randomization method. Moreover, no trial provided detailed information on the concealment of treatment allocation and blinding. Third, the course of treatment and dosage of DCVB6 injection varied between trials, and the chemotherapy regimens among the trials were also different. Furthermore, there was clinical heterogeneity between trials which might have affected the results. Fourth, trials included in the study were all published in Chinese literature. Potential selection and publication biases were inevitable due to the lack of gray literature.

In 1997, LeLorier et al. [34] published a study in The New England Journal of Medicine comparing the consistency of large RCTs with the conclusions of meta-analyses. The results indicated that the positive and negative predictive values of meta-analyses of large-scale RCTs were 68% and 67%, respectively. Therefore, meta-analyses cannot completely replace well-designed clinical trials with large sample sizes. The results of well-designed clinical trials and meta-analyses should be compared objectively and comprehensively to improve the accuracy of the conclusions. Hence, evidence-based medical data to enhance the efficacy of chemotherapy and reduce the side effects of DCVB6 injection in NSCLC are still inadequate. Only further high-quality multicenter clinical trials with large sample sizes can generate more accurate clinical evidence.

## 5. Conclusions

In conclusion, we found that DCVB6 injection combined with chemotherapy can improve ORR, KPS, and clinical symptoms and reduce chemotherapy toxicity when compared with chemotherapy alone for NSCLC patients. However, this conclusion should be interpreted with caution because of the poor quality of the trials. There is an urgent need for high-quality research that can be precisely evaluated to support this conclusion, particularly regarding the descriptions of methodologies and study processes.

## Figures and Tables

**Table 1:** Summary of trial characteristics included in the meta-analysis.

Study	N(T/C)	Age		Physical	Clinical stage		Interventions		Methods score	Outcomes
		T	C		T	C	T	C		
Wen et al., 2012[15]	34/34	32-70(58)		KPS≥60	III(25),IV(43)		DCVB6 50 ml/day, for 10 days + control	DP	8	①③④⑥
Song et al., 2012[16]	29/29	35-81(52.6)	28-85(53.4)	KPS>60	IIIb(20),IV(9)	IIIb(18),V(11)	DCVB6 30 ml/day, for 14 days + control	TP	7	①②
Zhang, 2012[17]	35/30	53-78(64)	51-76(62.5)	KPS≥60	IIIb(22),IV(13)	IIIb(19),IV(11)	DCVB6 30 ml/day, for 14 days + control	TP	9	①②④⑤⑥
Cao et al., 2013[12]	40/39	45-73(67)	47-75(65)	KPS≥60	IIIb(18),IV(22)	IIIb(20),IV(19)	DCVB6 40 ml/day, for 14 days + control	GP	8	①
Dong et al., 2013[13]	60/60	41-70(57.13)	40-72(56.27)	KPS≥60	III(22),IV(38)	IIIb(26),IV(34)	DCVB6 50 ml/day, for 10 days + control	DP	7	①②③④⑥
Wang et al., 2013[14]	42/38	32-70(42)	31-58(44)	KPS≥60	III(24),IV(18)	III(22),IV(16)	DCVB6 50 ml/day, for 14 days + control	NP	8	①④⑥
Sun et al., 2013[18]	36/32	49-68(51)	52-67(53)	KPS≥60	NR		DCVB6 50 ml/day, for 10 days + control	DP	8	①②④⑤⑥
Yuan et al., 2013[19]	27/26	61	60	KPS>70	NR		DCVB6 40 ml/day, for 14 days + control	DP, TP, GP or NP	9	①②
Zeng et al., 2014[20]	21/21	38-76(56)	40-74(58)	KPS>60	III(9),IVa(12)	III(8),IVa(13)	DCVB6 50 ml/day, for 14 days + control	DP	9	②④⑤⑥
Jia et al., 2014[21]	19/16	45-63		NR	NR		DCVB6 20 ml/day, for 15 days + control	DP	7	①④⑤
Dong et al., 2014[22]	48/46	26-75(56.5)	38-74(57.3)	KPS>30	III(21),IV(27)	III(19),IV(27)	DCVB6 50 ml/day, for 10 days + control	GP	8	①②③④⑤⑥
Chen et al., 2015[23]	42/42	45-78	43-76	KPS≥60	IIIb(30),IV(12)	IIIb(30),IV(12)	DCVB6 40 ml/day, for 10 days + control	GP	8	①②④⑤⑥
Wang et al., 2015[24]	76/78	71.3	72.4	KPS≥60	III(80),IV(74)		DCVB6 40 ml/day, for 14 days + control	AP	9	①②④⑥
Liu et al., 2015[25]	49/49	28-74(52.1)	30-75(52.6)	NR	III(27),IV(22)	III(25),IV(24)	DCVB6 25 ml/day, for 14 days + control	GP	8	①②③④⑤⑥
Li, 2015[26]	36/36	40-80(56.9)		NR	IIIb(42),IV(30)		DCVB6 50 ml/day, for 15 days + control	TP	9	①
Hu et al., 2016[27]	28/28	38-72(55)	39-73(56)	KPS≥70	NR		DCVB6 40 ml/day, for 10 days + control	GP	8	①②④⑤⑥
Li et al., 2016[28]	40/40	20-74(54.35)	28-76(54.39)	KPS≥70	IIIb(27),IV(13)	IIIb(25),IV(15)	DCVB6 30 ml/day, for 14 days + control	GP	8	①②④⑤⑥
Du et al., 2017[29]	30/30	52-78(65.27)	51-76(65.29)	NR	NR		DCVB6 50 ml/day, for 10 days + control	GP	8	①
Chen, 2017[30]	31/31	40-71(55.27)	41-70(54.52)	KPS≥60	III(12),IV(19)	III(13),IV(18)	DCVB6 50 ml/day, for 10 days + control	NP	8	①③④⑥

DP: docetaxel + cisplatin, TP: paclitaxel + cisplatin, GP: gemcitabine + cisplatin, NP: navelbine + cisplatin, AP: pemetrexed + cisplatin, ① ORR, ② KPS, ③ clinical symptom, ④ WBC toxicity, ⑤ platelet toxicity, and ⑥ vomiting.

## Supplementary Materials

Supplementary Materials The data abstraction form for methodological quality including 9 questions and the specific criteria are shown in Table S1. The subgroup analysis of DCVB6 injection for different doses and durations has been carried out and the forest plots of ORR, KPS, WBC toxicity, and vomiting are shown in Figures S1-S8. Figure S1: the subgroup analysis of ORR for different doses of DCVB6 injection. There was no differential effect (*P*=0.86) of DCVB6 injection on ORR between the subgroup of low dose (RR=1.60, 95% CI 1.37-1.86) and high dose (RR=1.56, 95% CI 1.28-1.90). Figure S2: the subgroup analysis of KPS for different doses of DCVB6 injection. There was no differential effect (*P*=0.57) of DCVB6 injection on KPS between the subgroup of low dose (RR=1.75, 95% CI 1.40-2.19) and high dose (RR=1.59, 95% CI 1.23-2.05). Figure S3: the subgroup analysis of ORR for different durations of DCVB6 injection. There was no differential effect (*P*=0.22) of DCVB6 injection on ORR between the subgroup of short duration (RR=1.44, 95% CI 1.17-1.76) and long duration (RR=1.68, 95% CI 1.45-1.96). Figure S4: the subgroup analysis of KPS for different durations of DCVB6 injection. There was no differential effect (*P*=0.61) of DCVB6 injection on KPS between the subgroup of short duration (RR=1.61, 95% CI 1.28-2.03) and long duration (RR=1.76, 95% CI 1.38-2.25). Figure S5: the subgroup analysis of WBC toxicity for different doses of DCVB6 injection. There was no differential effect (*P*=0.98) of DCVB6 injection on WBC toxicity between the subgroup of low dose (RR=0.36, 95% CI 0.24-0.55) and high dose (RR=0.36, 95% CI 0.23-0.56). Figure S6: the subgroup analysis of vomiting for different doses of DCVB6 injection. There was no differential effect (*P*=0.27) of DCVB6 injection on vomiting between the subgroup of low dose (RR=0.39, 95% CI 0.23-0.67) and high dose (RR=0.57, 95% CI 0.40-0.81). Figure S7: the subgroup analysis of WBC toxicity for different durations of DCVB6 injection. There was no differential effect (*P*=0.70) of DCVB6 injection on WBC toxicity between the subgroup of short duration (RR=0.38, 95% CI 0.25-0.57) and long duration (RR=0.34, 95% CI 0.22-0.53). Figure S8: the subgroup analysis of vomiting for different durations of DCVB6 injection. There was no differential effect (*P*=0.51) of DCVB6 injection on vomiting between the subgroup of short duration (RR=0.46, 95% CI 0.32-0.68) and long duration (RR=0.57, 95% CI 0.35-0.91).
