# Supplementary material for: Disodium Cantharidinate and Vitamin B6 Injection Adjunct with Platinum-Based Chemotherapy for the Treatment of Advanced Non-Small-Cell Lung Cancer: A Meta-Analysis
Source: Evid Based Complement Alternat Med. 2019 Mar 12;2019:9386273. doi: 10.1155/2019/9386273 (PMC6434307; doi:10.1155/2019/9386273)
Supplement: Supplementary Materials — The data abstraction form for methodological quality including 9 questions and the specific criteria are shown in Table S1. The subgroup analysis of DCVB6 injection for different doses and durations has been carried out and the forest plots of ORR, KPS, WBC toxicity, and vomiting are shown in Figures S1-S8. Figure S1: the subgroup analysis of ORR for different doses of DCVB6 injection. There was no differential effect (P=0.86) of DCVB6 injection on ORR between the subgroup of low dose (RR=1.60, 95% CI 1.37-1.86) and high dose (RR=1.56, 95% CI 1.28-1.90). Figure S2: the subgroup analysis of KPS for different doses of DCVB6 injection. There was no differential effect (P=0.57) of DCVB6 injection on KPS between the subgroup of low dose (RR=1.75, 95% CI 1.40-2.19) and high dose (RR=1.59, 95% CI 1.23-2.05). Figure S3: the subgroup analysis of ORR for different durations of DCVB6 injection. There was no differential effect (P=0.22) of DCVB6 injection on ORR between the subgroup of short duration (RR=1.44, 95% CI 1.17-1.76) and long duration (RR=1.68, 95% CI 1.45-1.96). Figure S4: the subgroup analysis of KPS for different durations of DCVB6 injection. There was no differential effect (P=0.61) of DCVB6 injection on KPS between the subgroup of short duration (RR=1.61, 95% CI 1.28-2.03) and long duration (RR=1.76, 95% CI 1.38-2.25). Figure S5: the subgroup analysis of WBC toxicity for different doses of DCVB6 injection. There was no differential effect (P=0.98) of DCVB6 injection on WBC toxicity between the subgroup of low dose (RR=0.36, 95% CI 0.24-0.55) and high dose (RR=0.36, 95% CI 0.23-0.56). Figure S6: the subgroup analysis of vomiting for different doses of DCVB6 injection. There was no differential effect (P=0.27) of DCVB6 injection on vomiting between the subgroup of low dose (RR=0.39, 95% CI 0.23-0.67) and high dose (RR=0.57, 95% CI 0.40-0.81). Figure S7: the subgroup analysis of WBC toxicity for different durations of DCVB6 injection. There was no differential effe [file 9386273.f1.pdf]

Supplementary Materials

Table S1: Criteria used to assess methodological quality*

	Score		
	**0**	**1**	**2**
Randomization	…	Not concealed or not sure	Concealed randomization
Blinding	Not blinded	…	Adjudicators blinded
Analysis	Other	…	Intention to treat
Patient selection	Selected patients or unable to tell	Consecutive eligible patients	…
Comparability of groups at baseline	No or not sure	Yes	…
Extent of follow-up	<100%	100%	…
Treatment protocol	Poorly described	Reproducibly described	…
Cointerventions	Not described	Described but not equal or not sure	Well described and all equal
Outcomes	Not described	Partially described	Objectively defined

*The first 3questions and the last 2 questions had possible scores of 0, 1, or 2. The middle 3 questions had possible scores of 0 or 1. The highest possible score was 14. Ellipses indicate data not applicable.

Study or Subgroup	Experimental Events	Experimental Total	Control Events	Control Total	Weight	Risk Ratio M-H, Fixed, 95% CI
**3.10.1 < 50 ml/day**						
Cao et al. 2013	29	40	19	39	13.8%	1.49 [1.02, 2.16]
Chen et al. 2015	20	42	13	42	9.4%	1.54 [0.89, 2.67]
Hu et al. 2016	10	28	8	28	5.8%	1.25 [0.58, 2.69]
Jia et al. 2014	11	19	3	16	2.3%	3.09 [1.04, 9.18]
Li et al. 2016	26	40	14	40	10.1%	1.86 [1.15, 3.00]
Liu et al. 2015	26	49	16	49	11.5%	1.63 [1.00, 2.63]
Song et al. 2012	22	29	18	29	12.9%	1.22 [0.86, 1.74]
Wang et al. 2015	54	76	32	78	22.7%	1.73 [1.28, 2.34]
Yuan et al. 2013	7	27	4	26	2.9%	1.69 [0.56, 5.08]
Zhang 2012	19	35	11	30	8.5%	1.48 [0.85, 2.59]
**Subtotal (95% CI)**		**385**		**377**	**100.0%**	**1.60 [1.37, 1.86]**
Total events	224		138			
Heterogeneity: Chi² = 4.92, df = 9 (P = 0.84); I² = 0%						
Test for overall effect: Z = 5.96 (P < 0.00001)						
**3.10.2 ≥ 50 ml/day**						
Chen 2017	14	31	10	31	10.0%	1.40 [0.74, 2.66]
Dong et al. 2013	24	60	20	60	20.1%	1.20 [0.75, 1.93]
Dong et al. 2014	23	48	13	46	13.3%	1.70 [0.98, 2.93]
Du et al. 2017	16	30	8	30	8.0%	2.00 [1.01, 3.95]
Li 2015	20	36	10	36	10.0%	2.00 [1.10, 3.65]
Sun et al. 2013	14	36	11	32	11.7%	1.13 [0.60, 2.12]
Wang et al. 2013	26	42	12	38	12.7%	1.96 [1.16, 3.31]
Wen et al. 2012	21	34	14	34	14.1%	1.50 [0.93, 2.43]
**Subtotal (95% CI)**		**317**		**307**	**100.0%**	**1.56 [1.28, 1.90]**
Total events	158		98			
Heterogeneity: Chi² = 4.30, df = 7 (P = 0.75); I² = 0%						
Test for overall effect: Z = 4.43 (P < 0.00001)						

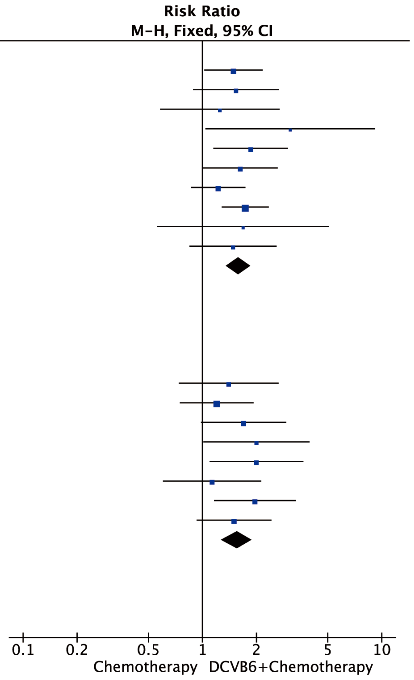

Test for subgroup differences: Chi² = 0.03, df = 1 (P = 0.86), I² = 0%

Figure S1: Subgroup analysis of ORR for different doses of DCVB6 injection

Study or Subgroup	Experimental Events	Experimental Total	Control Events	Control Total	Weight	Risk Ratio M-H, Fixed, 95% CI
**3.11.1 < 50 ml/day**						
Chen et al. 2015	21	42	13	42	16.9%	1.62 [0.94, 2.78]
Hu et al. 2016	17	28	9	28	11.7%	1.89 [1.02, 3.49]
Li et al. 2016	20	40	12	40	15.6%	1.67 [0.95, 2.93]
Liu et al. 2015	24	49	13	49	16.9%	1.85 [1.07, 3.19]
Song et al. 2012	14	29	10	29	13.0%	1.40 [0.75, 2.62]
Wang et al. 2015	7	28	5	39	5.4%	1.95 [0.69, 5.52]
Yuan et al. 2013	15	27	10	26	13.3%	1.44 [0.80, 2.61]
Zhang 2012	17	35	5	30	7.0%	2.91 [1.22, 6.95]
**Subtotal (95% CI)**		**278**		**283**	**100.0%**	**1.75 [1.40, 2.19]**
Total events	135		77			
Heterogeneity: Chi² = 2.47, df = 7 (P = 0.93); I² = 0%						
Test for overall effect: Z = 4.92 (P < 0.00001)						
**3.11.2 ≥ 50 ml/day**						
Dong et al. 2013	30	60	14	60	25.7%	2.14 [1.27, 3.62]
Dong et al. 2014	23	48	12	46	22.5%	1.84 [1.04, 3.24]
Sun et al. 2013	21	36	19	32	37.0%	0.98 [0.66, 1.46]
Zeng et al. 2014	14	21	8	21	14.7%	1.75 [0.94, 3.26]
**Subtotal (95% CI)**		**165**		**159**	**100.0%**	**1.59 [1.23, 2.05]**
Total events	88		53			
Heterogeneity: Chi² = 7.19, df = 3 (P = 0.07); I² = 58%						
Test for overall effect: Z = 3.51 (P = 0.0004)						

Test for subgroup differences: Chi² = 0.33, df = 1 (P = 0.57), I² = 0%

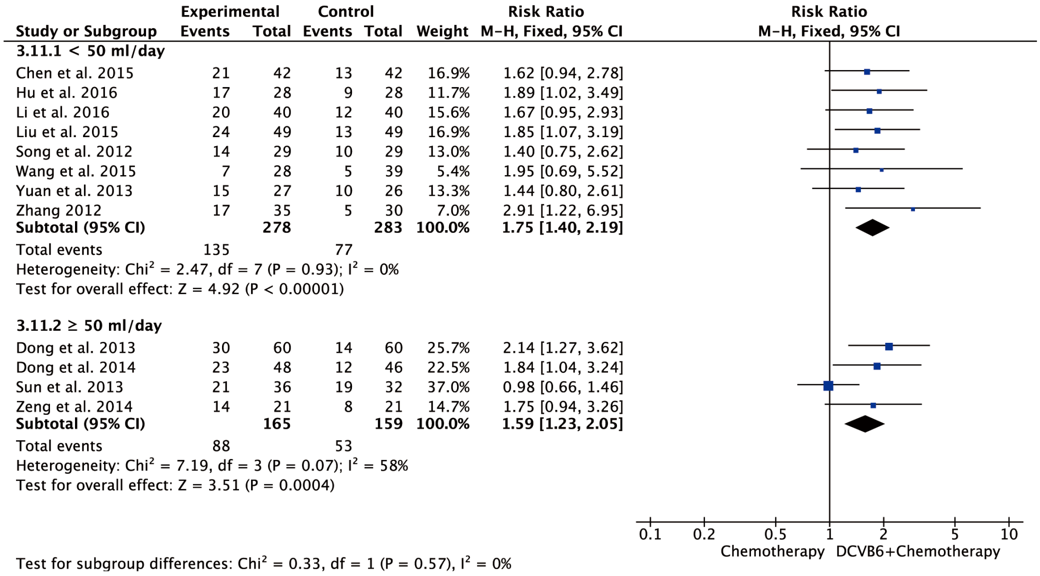

Figure S2: Subgroup analysis of KPS for different doses of DCVB6 injection

Study or Subgroup	Experimental Events	Experimental Total	Control Events	Control Total	Weight	Risk Ratio M-H, Fixed, 95% CI
**3.6.1 ≤ 10 days**						
Chen 2017	14	31	10	31	10.2%	1.40 [0.74, 2.66]
Chen et al. 2015	20	42	13	42	13.3%	1.54 [0.89, 2.67]
Dong et al. 2013	24	60	20	60	20.4%	1.20 [0.75, 1.93]
Dong et al. 2014	23	48	13	46	13.6%	1.70 [0.98, 2.93]
Du et al. 2017	16	30	8	30	8.2%	2.00 [1.01, 3.95]
Hu et al. 2016	10	28	8	28	8.2%	1.25 [0.58, 2.69]
Sun et al. 2013	14	36	11	32	11.9%	1.13 [0.60, 2.12]
Wen et al. 2012	21	34	14	34	14.3%	1.50 [0.93, 2.43]
**Subtotal (95% CI)**		**309**		**303**	**100.0%**	**1.44 [1.17, 1.76]**
Total events	142		97			
Heterogeneity: Chi² = 2.59, df = 7 (P = 0.92); I² = 0%						
Test for overall effect: Z = 3.49 (P = 0.0005)						
**3.6.2 > 10 days**						
Cao et al. 2013	29	40	19	39	13.7%	1.49 [1.02, 2.16]
Jia et al. 2014	11	19	3	16	2.3%	3.09 [1.04, 9.18]
Li 2015	20	36	10	36	7.1%	2.00 [1.10, 3.65]
Li et al. 2016	26	40	14	40	10.0%	1.86 [1.15, 3.00]
Liu et al. 2015	26	49	16	49	11.4%	1.63 [1.00, 2.63]
Song et al. 2012	22	29	18	29	12.8%	1.22 [0.86, 1.74]
Wang et al. 2013	26	42	12	38	9.0%	1.96 [1.16, 3.31]
Wang et al. 2015	54	76	32	78	22.5%	1.73 [1.28, 2.34]
Yuan et al. 2013	7	27	4	26	2.9%	1.69 [0.56, 5.08]
Zhang 2012	19	35	11	30	8.4%	1.48 [0.85, 2.59]
**Subtotal (95% CI)**		**393**		**381**	**100.0%**	**1.68 [1.45, 1.96]**
Total events	240		139			
Heterogeneity: Chi² = 5.85, df = 9 (P = 0.75); I² = 0%						
Test for overall effect: Z = 6.74 (P < 0.00001)						

Test for subgroup differences: Chi² = 1.49, df = 1 (P = 0.22), I² = 32.8%

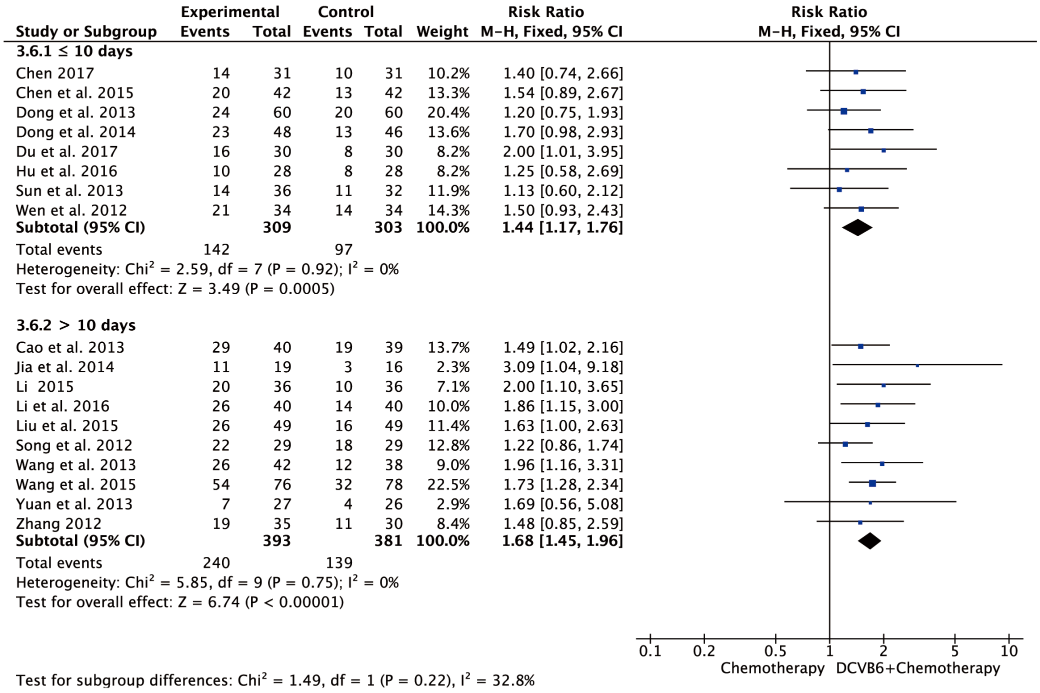

Figure S3: Subgroup analysis of ORR for different durations of DCVB6 injection

Study or Subgroup	Experimental Events	Experimental Total	Control Events	Control Total	Weight	Risk Ratio M-H, Fixed, 95% CI
**3.7.1 ≤ 10 days**						
Chen et al. 2015	21	42	13	42	19.0%	1.62 [0.94, 2.78]
Dong et al. 2013	30	60	14	60	20.5%	2.14 [1.27, 3.62]
Dong et al. 2014	23	48	12	46	17.9%	1.84 [1.04, 3.24]
Hu et al. 2016	17	28	9	28	13.2%	1.89 [1.02, 3.49]
Sun et al. 2013	21	36	19	32	29.4%	0.98 [0.66, 1.46]
**Subtotal (95% CI)**		**214**		**208**	**100.0%**	**1.61 [1.28, 2.03]**
Total events	112		67			
Heterogeneity: $Chi^2 = 7.55$, df = 4 (P = 0.11); $I^2 = 47\%$						
Test for overall effect: Z = 4.04 (P < 0.0001)						
**3.7.2 > 10 days**						
Li et al. 2016	20	40	12	40	19.1%	1.67 [0.95, 2.93]
Liu et al. 2015	24	49	13	49	20.7%	1.85 [1.07, 3.19]
Song et al. 2012	14	29	10	29	15.9%	1.40 [0.75, 2.62]
Wang et al. 2015	7	28	5	39	6.7%	1.95 [0.69, 5.52]
Yuan et al. 2013	15	27	10	26	16.2%	1.44 [0.80, 2.61]
Zeng et al. 2014	14	21	8	21	12.7%	1.75 [0.94, 3.26]
Zhang 2012	17	35	5	30	8.6%	2.91 [1.22, 6.95]
**Subtotal (95% CI)**		**229**		**234**	**100.0%**	**1.76 [1.38, 2.25]**
Total events	111		63			
Heterogeneity: $Chi^2 = 2.34$, df = 6 (P = 0.89); $I^2 = 0\%$						
Test for overall effect: Z = 4.50 (P < 0.00001)						

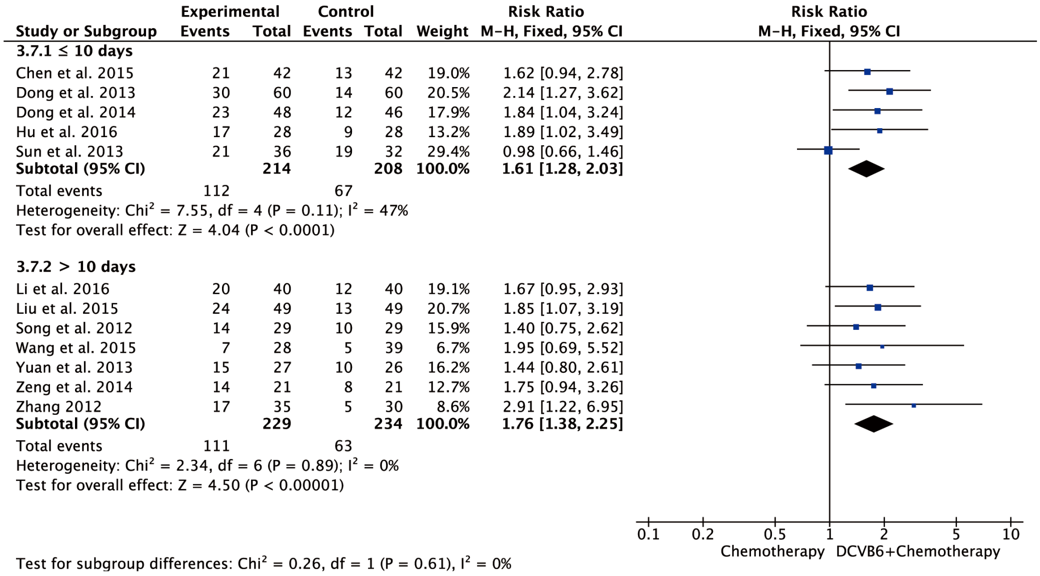

Test for subgroup differences: $Chi^2 = 0.26$, df = 1 (P = 0.61), $I^2 = 0\%$

Figure S4: Subgroup analysis of KPS for different durations of DCVB6 injection

Study or Subgroup	Experimental Events	Experimental Total	Control Events	Control Total	Weight	Risk Ratio M-H, Fixed, 95% CI
**3.12.1 < 50 ml/day**						
Chen et al. 2015	3	42	5	42	7.3%	0.60 [0.15, 2.35]
Hu et al. 2016	5	28	12	28	17.6%	0.42 [0.17, 1.03]
Jia et al. 2014	1	19	6	16	9.6%	0.14 [0.02, 1.05]
Li et al. 2016	2	40	5	40	7.3%	0.40 [0.08, 1.94]
Liu et al. 2015	10	49	27	49	39.7%	0.37 [0.20, 0.68]
Wang et al. 2015	1	38	4	39	5.8%	0.26 [0.03, 2.19]
Zhang 2012	3	35	8	30	12.7%	0.32 [0.09, 1.10]
**Subtotal (95% CI)**		**251**		**244**	**100.0%**	**0.36 [0.24, 0.55]**
Total events	25		67			
Heterogeneity: $Chi^2 = 1.63$, df = 6 (P = 0.95); $I^2 = 0\%$						
Test for overall effect: Z = 4.87 (P < 0.00001)						
**3.12.2 ≥ 50 ml/day**						
Chen 2017	1	31	12	31	19.3%	0.08 [0.01, 0.60]
Dong et al. 2013	2	60	6	60	9.6%	0.33 [0.07, 1.59]
Dong et al. 2014	10	48	19	46	31.1%	0.50 [0.26, 0.97]
Sun et al. 2013	3	36	8	32	13.6%	0.33 [0.10, 1.15]
Wang et al. 2013	4	42	9	38	15.2%	0.40 [0.13, 1.20]
Wen et al. 2012	3	34	7	34	11.2%	0.43 [0.12, 1.52]
Zeng et al. 2014	0	21	0	21		Not estimable
**Subtotal (95% CI)**		**272**		**262**	**100.0%**	**0.36 [0.23, 0.56]**
Total events	23		61			
Heterogeneity: $Chi^2 = 3.28$, df = 5 (P = 0.66); $I^2 = 0\%$						
Test for overall effect: Z = 4.56 (P < 0.00001)						

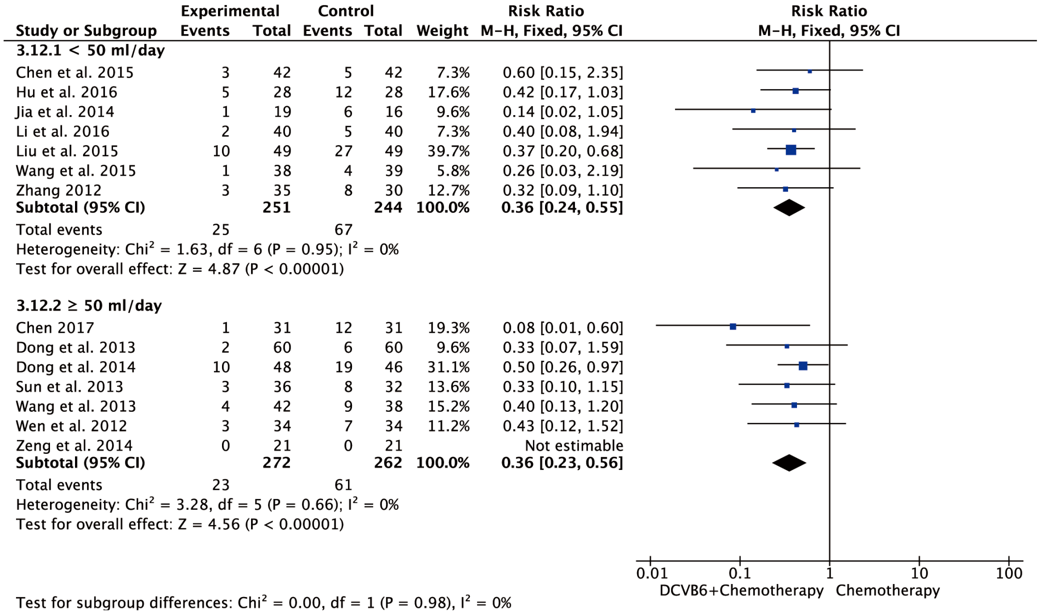

Test for subgroup differences: $Chi^2 = 0.00$, df = 1 (P = 0.98), $I^2 = 0\%$

Figure S5: Subgroup analysis of WBC toxicity for different doses of DCVB6 injection

Study or Subgroup	Experimental Events	Experimental Total	Control Events	Control Total	Weight	Risk Ratio M-H, Fixed, 95% CI
**3.13.1 < 50 ml/day**						
Chen et al. 2015	1	42	3	42	7.5%	0.33 [0.04, 3.08]
Hu et al. 2016	3	28	10	28	24.9%	0.30 [0.09, 0.98]
Li et al. 2016	1	40	2	40	5.0%	0.50 [0.05, 5.30]
Liu et al. 2015	8	49	18	49	44.8%	0.44 [0.21, 0.92]
Wang et al. 2015	1	38	4	39	9.8%	0.26 [0.03, 2.19]
Zhang 2012	2	35	3	30	8.0%	0.57 [0.10, 3.20]
**Subtotal (95% CI)**		**232**		**228**	**100.0%**	**0.39 [0.23, 0.67]**
Total events	16		40			
Heterogeneity: Chi² = 0.70, df = 5 (P = 0.98); I² = 0%						
Test for overall effect: Z = 3.42 (P = 0.0006)						
**3.13.2 ≥ 50 ml/day**						
Chen 2017	3	31	6	31	9.4%	0.50 [0.14, 1.82]
Dong et al. 2013	14	60	26	60	40.6%	0.54 [0.31, 0.93]
Dong et al. 2014	6	48	15	46	23.9%	0.38 [0.16, 0.90]
Sun et al. 2013	2	36	3	32	5.0%	0.59 [0.11, 3.32]
Wang et al. 2013	10	42	10	38	16.4%	0.90 [0.42, 1.93]
Wen et al. 2012	2	34	3	34	4.7%	0.67 [0.12, 3.74]
Zeng et al. 2014	0	21	0	21		Not estimable
**Subtotal (95% CI)**		**272**		**262**	**100.0%**	**0.57 [0.40, 0.81]**
Total events	37		63			
Heterogeneity: Chi² = 2.37, df = 5 (P = 0.80); I² = 0%						
Test for overall effect: Z = 3.12 (P = 0.002)						

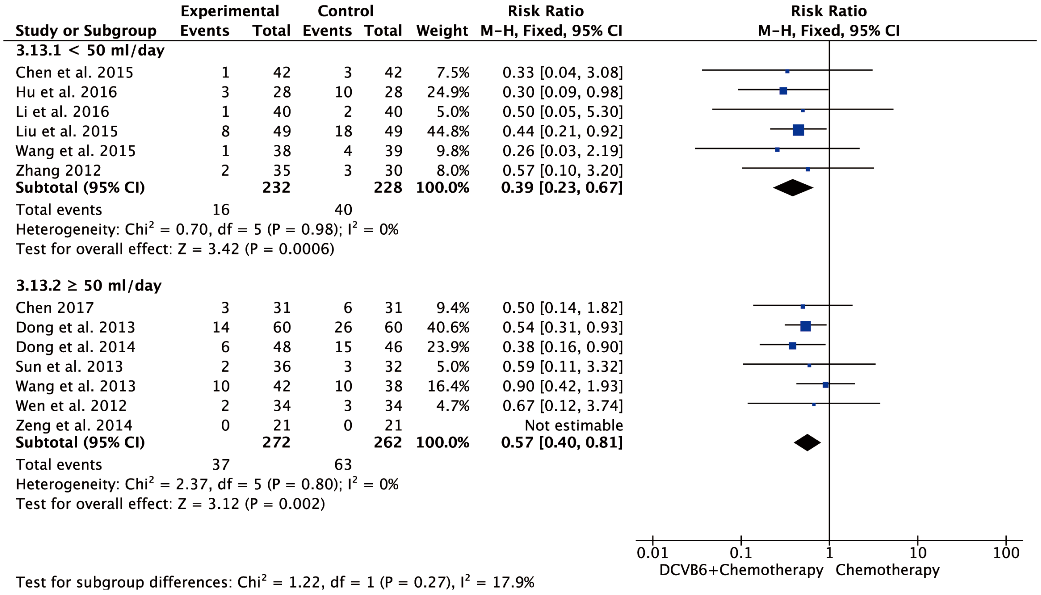

Test for subgroup differences: Chi² = 1.22, df = 1 (P = 0.27), I² = 17.9%

Figure S6: Subgroup analysis of vomiting for different doses of DCVB6 injection

Study or Subgroup	Experimental Events	Experimental Total	Control Events	Control Total	Weight	Risk Ratio M-H, Fixed, 95% CI
**3.8.1 ≤ 10 days**						
Chen 2017	1	31	12	31	17.2%	0.08 [0.01, 0.60]
Chen et al. 2015	3	42	5	42	7.2%	0.60 [0.15, 2.35]
Dong et al. 2013	2	60	6	60	8.6%	0.33 [0.07, 1.59]
Dong et al. 2014	10	48	19	46	27.8%	0.50 [0.26, 0.97]
Hu et al. 2016	5	28	12	28	17.2%	0.42 [0.17, 1.03]
Sun et al. 2013	3	36	8	32	12.1%	0.33 [0.10, 1.15]
Wen et al. 2012	3	34	7	34	10.0%	0.43 [0.12, 1.52]
**Subtotal (95% CI)**		**279**		**273**	**100.0%**	**0.38 [0.25, 0.57]**
Total events	27		69			
Heterogeneity: Chi² = 3.55, df = 6 (P = 0.74); I² = 0%						
Test for overall effect: Z = 4.68 (P < 0.00001)						
**3.8.2 > 10 days**						
Jia et al. 2014	1	19	6	16	10.8%	0.14 [0.02, 1.05]
Li et al. 2016	2	40	5	40	8.3%	0.40 [0.08, 1.94]
Liu et al. 2015	10	49	27	49	44.6%	0.37 [0.20, 0.68]
Wang et al. 2013	4	42	9	38	15.6%	0.40 [0.13, 1.20]
Wang et al. 2015	1	38	4	39	6.5%	0.26 [0.03, 2.19]
Zeng et al. 2014	0	21	0	21		Not estimable
Zhang 2012	3	35	8	30	14.2%	0.32 [0.09, 1.10]
**Subtotal (95% CI)**		**244**		**233**	**100.0%**	**0.34 [0.22, 0.53]**
Total events	21		59			
Heterogeneity: Chi² = 1.03, df = 5 (P = 0.96); I² = 0%						
Test for overall effect: Z = 4.76 (P < 0.00001)						

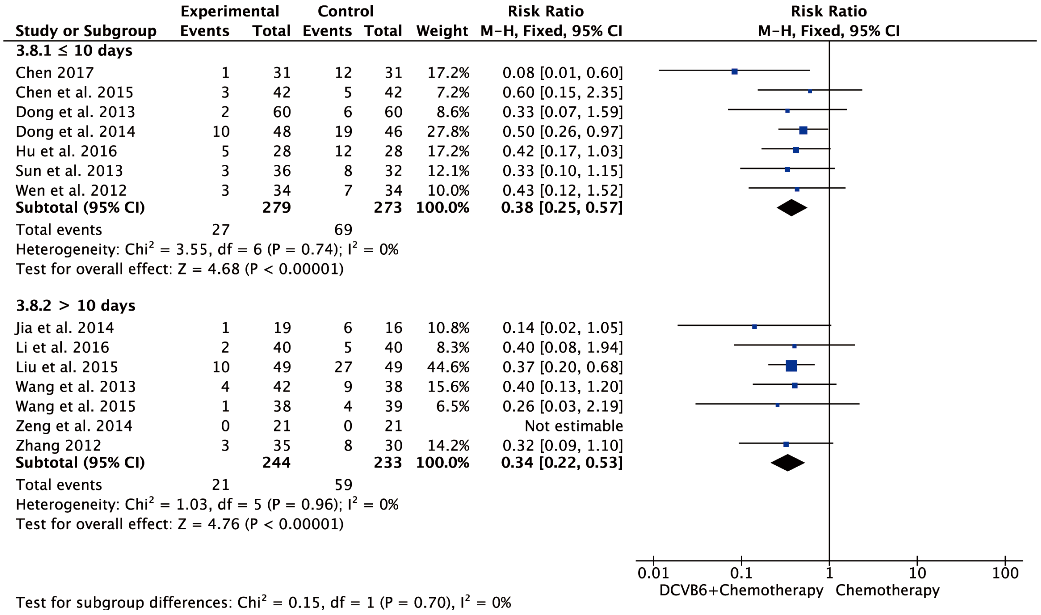

Test for subgroup differences: Chi² = 0.15, df = 1 (P = 0.70), I² = 0%

Figure S7: Subgroup analysis of WBC toxicity for different durations of DCVB6 injection

Study or Subgroup	Experimental Events	Experimental Total	Control Events	Control Total	Weight	Risk Ratio M-H, Fixed, 95% CI
**3.9.1 ≤ 10 days**						
Chen 2017	3	31	6	31	9.0%	0.50 [0.14, 1.82]
Chen et al. 2015	1	42	3	42	4.5%	0.33 [0.04, 3.08]
Dong et al. 2013	14	60	26	60	39.1%	0.54 [0.31, 0.93]
Dong et al. 2014	6	48	15	46	23.0%	0.38 [0.16, 0.90]
Hu et al. 2016	3	28	10	28	15.0%	0.30 [0.09, 0.98]
Sun et al. 2013	2	36	3	32	4.8%	0.59 [0.11, 3.32]
Wen et al. 2012	2	34	3	34	4.5%	0.67 [0.12, 3.74]
**Subtotal (95% CI)**		**279**		**273**	**100.0%**	**0.46 [0.32, 0.68]**
Total events	31		66			
Heterogeneity: Chi² = 1.35, df = 6 (P = 0.97); I² = 0%						
Test for overall effect: Z = 3.98 (P < 0.0001)						
**3.9.2 > 10 days**						
Li et al. 2016	1	40	2	40	5.3%	0.50 [0.05, 5.30]
Liu et al. 2015	8	49	18	49	47.8%	0.44 [0.21, 0.92]
Wang et al. 2013	10	42	10	38	27.9%	0.90 [0.42, 1.93]
Wang et al. 2015	1	38	4	39	10.5%	0.26 [0.03, 2.19]
Zeng et al. 2014	0	21	0	21		Not estimable
Zhang 2012	2	35	3	30	8.6%	0.57 [0.10, 3.20]
**Subtotal (95% CI)**		**225**		**217**	**100.0%**	**0.57 [0.35, 0.91]**
Total events	22		37			
Heterogeneity: Chi² = 2.42, df = 4 (P = 0.66); I² = 0%						
Test for overall effect: Z = 2.34 (P = 0.02)						

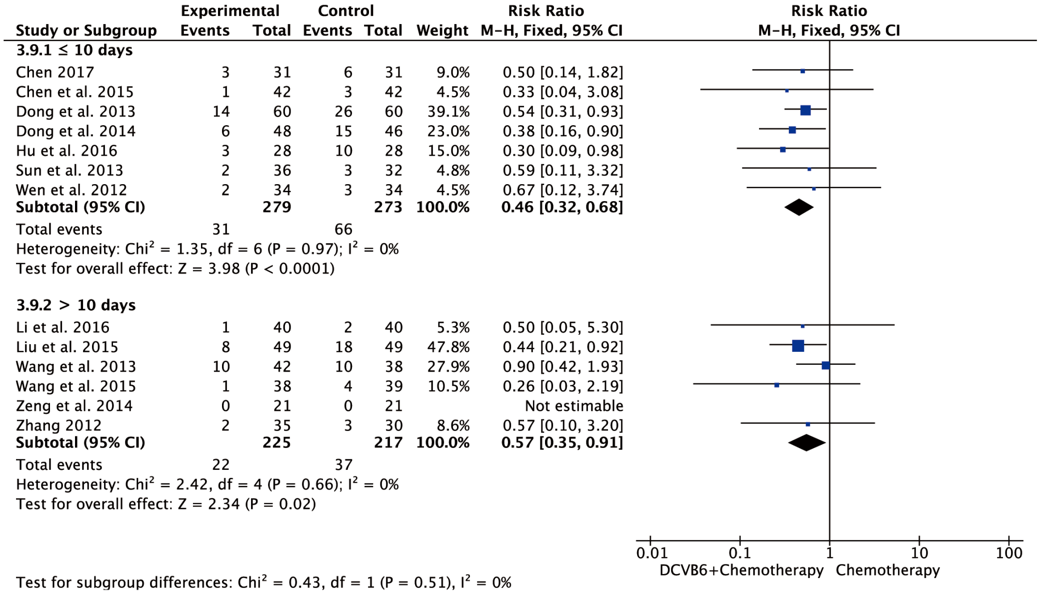

Test for subgroup differences: Chi² = 0.43, df = 1 (P = 0.51), I² = 0%

Figure S8: Subgroup analysis of vomiting for different durations of DCVB6 injection
